# Pro-Inflammatory and Pro-Oxidant Status of Pancreatic Islet *In Vitro* Is Controlled by TLR-4 and HO-1 Pathways

**DOI:** 10.1371/journal.pone.0107656

**Published:** 2014-10-24

**Authors:** Kevin Vivot, Allan Langlois, William Bietiger, Stéphanie Dal, Elodie Seyfritz, Michel Pinget, Nathalie Jeandidier, Elisa Maillard, Jean-Pierre Gies, Séverine Sigrist

**Affiliations:** 1 DIATHEC, EA 7294, Centre Européen d'Etude du Diabète, Université de Strasbourg, Fédération de Médecine Translationnelle, Strasbourg, France; 2 Structure d'Endocrinologie, Diabète –Nutrition et Addictologie, Pôle NUDE, Hôpitaux Universitaires de Strasbourg, (HUS), Strasbourg, France; 3 UMR 7034 CNRS, Faculté de Pharmacie, Université de Strasbourg, Illkirch, France; Fundação Oswaldo Cruz, Brazil

## Abstract

Since their isolation until implantation, pancreatic islets suffer a major stress leading to the activation of inflammatory reactions. The maintenance of controlled inflammation is essential to preserve survival and function of the graft. Identification and targeting of pathway(s) implicated in post-transplant detrimental inflammatory events, is mandatory to improve islet transplantation success. We sought to characterize the expression of the pro-inflammatory and pro-oxidant mediators during islet culture with a focus on Heme oxygenase (HO-1) and Toll-like receptors-4 signaling pathways. Rat pancreatic islets were isolated and pro-inflammatory and pro-oxidant status were evaluated after 0, 12, 24 and 48 hours of culture through TLR-4, HO-1 and cyclooxygenase-2 (COX-2) expression, CCL-2 and IL-6 secretion, ROS (Reactive Oxygen Species) production (Dihydroethidine staining, DHE) and macrophages migration. To identify the therapeutic target, TLR4 inhibition (CLI-095) and HO-1 activation (cobalt protoporphyrin,CoPP) was performed. Activation of NFκB signaling pathway was also investigated. After isolation and during culture, pancreatic islet exhibited a proinflammatory and prooxidant status (increase levels of TLR-4, COX-2, CCL-2, IL-6, and ROS). Activation of HO-1 or inhibition of TLR-4 decreased inflammatory status and oxidative stress of islets. Moreover, the overexpression of HO-1 induced NFκB phosphorylation while the inhibition of TLR-4 had no effect NFκB activation. Finally, inhibition of pro-inflammatory pathway induced a reduction of macrophages migration. These data demonstrated that the TLR-4 signaling pathway is implicated in early inflammatory events leading to a pro-inflammatory and pro-oxidant status of islets *in vitro*. Moreover, these results provide the mechanism whereby the benefits of HO-1 target in TLR-4 signaling pathway. HO-1 could be then an interesting target to protect islets before transplantation.

## Introduction

Pancreatic islet transplantation is a non-invasive therapeutic option to replace the β-cell mass in type 1 diabetic patients with marked glycemic variability and/or severe and recurrent hypoglycemic episode [1]–[4]. During the last decade, important progress has been made in islet transplantation in clinical practice [5]–[7]. The Edmonton protocol has greatly increased the survival and initial function of transplanted islets. Significant improvement has occurred in the outcomes of clinical islet transplantation reflecting improvements in immunosuppression, and preparation of sufficient quantities of highly viable islets for transplantation [5], [8]–[10].

However, transplanted islets are particularly vulnerable in the first days after transplantation [11]. More than 50% of islets fail to engraft at this early phase, representing a major impediment for successful islet transplantation [12]. Moreover, even in optimal conditions, approximately 60% of transplanted syngeneic islet tissue is lost 3 days after transplantation [13]. Then, early graft loss in islet transplantation requires large amount of donors islets. [14], [15]. An early innate inflammatory reaction at the grafted site may be partly related to damage to islets during isolation [16] and strongly affects islet engraftment and survival after intrahepatic transplantation. This early reaction is triggered by ischemia-reperfusion injury and instant blood mediated inflammatory reaction (IBMIR) occurring hours and days after islet infusion. Evidence in both mouse model and in human counterpart suggests the involvement of coagulation, complement system, and proinflammatory chemokines/cytokines but macrophage mediated non specific inflammatory reaction could also contributes significantly to early dysfunction of islets grafts [8]. Mechanisms underlying early islet loss following transplantation remain poorly defined but apoptotic islet cell death associated with peri and intra-islet graft inflammation have been described [17], [18]. The process of isolation exposes islets to a number of stresses that can adversely affect cell survival [19]. Experiments suggest that islet isolation and culture induced a general inflammatory response [20]. The isolation procedure confers then a pro-inflammatory and pro-oxydant status of islets. In response, islets expressed a panel of pro-inflammatory mediators such as CCL2, IL-6, TNF-α [21]. CCL-2 is constitutively present in normal human islet cells, in the absence of an inflammatory infiltrate and CCL-2 secretion resulted as the more relevant factor for successful engraftment and long-lasting insulin independence. However, CCL-2 production by pancreatic islets is strongly increased by inflammatory cytokines, suggesting a possible involvement of this chemokine in the early inflammatory response [22]. Islet CCL2 release seems to be a sign of “inflammed” islets and a causal effect for developing detrimental proinflammatory conditions after transplant [23], [24]. In contrast, IL-6, TNF-α can stimulate the expression of cyclooxygenase-2 (COX-2), which is believed to be mediator of cytokine-induced islet damage [25], [26].

Moreover, Toll-like receptors (TLRs) transmitted signals activate innate immunity by inducing chemokine and cytokine release and through upregulating costimulatory molecule expression, among a multitude of other effects [27]. Recent studies revealed the importance of islet expressed TLRs, particularly TLR2 and TLR4, participating in the pathogenesis of autoimmune diabetes and allogeneic islet transplant rejection [28]–[30].TLR2 or TLR4 initiated a proinflammatory milieu, likely via chemokines and cytokine release at the graft site, associated with graft apoptosis and early graft [31]. TLR transmitted signals in the islets impact early islet engraftment has not been studied. However, a relationship between TLR and Heme oxygenase-1 (HO-1; heat shock protein hsp32) signaling pathway is established in hepatic ischemia-reperfusion injuries [32], and it is demonstrated that induction of HO-1 attenuates lipopolysaccharide-induced cyclooxygenase-2 expression in mouse brain endothelial cells [11]. In fact, HO-1 is among the most critical protective mechanisms activated during cellular stress, and it is thought to play a key role in maintaining anti-oxydant/oxidant imbalance [33]. Indeed, induction of HO-1 extends graft survival when islets are transplanted into the kidney capsule [8], [19]. Moreover, HO-1 decreases cytokine release and macrophage recruitment during the peri-transplant period [21].

Then, identification and targeting of pathway(s), playing a role as “master regulator(s)” in post-transplant detrimental inflammatory events, is now mandatory to improve islet transplantation success.

In this study, we hypothesized that the well-known benefits induce by HO-1 are due to the inhibition of the TLR-4 signaling pathway. To address this hypothesis, we first characterized the expression of the pro-inflammatory and anti-inflammatory mediators during islet culture. We next assessed the role of HO-1 and TLR-4 in pro-inflammatory status of islets and validate the consequence on macrophage migration.

## Materials and Methods

### Animals

Experiments were performed in accordance with the principles and guidelines of the French legislation on animal welfare (n° 2013-118) and in the strict accordance with the recommendations in the guide for the Care and Use of Laboratory Animals of the Nationals Institutes of Health. The protocol was approved by the Institutional Animal Care and Use Committee CREMEAS (n°AL/06/35/12/12). Wistar rats (250–300 g) were purchased from Depré Breeding (Elevage Depré,St Doulchar, France). Rats were housed in standard collective cages, in a temperature-controlled room (22±1°C) with a 12 h light/12 h darkness cycle. They were fed with SAFE-A04 (Villemoisson-sur-Orge, France). Food and water were available ad libitum. All surgery was performed under anesthesia induced by intraperitoneal injection at the dose of 100 µL/100 g of body weight of Imalgene (Ketamine, Merial, Lyon, France) supplemented with 2.7 mL Rompun (Xylazine 2%, Bayer, Puteaux, France). and all efforts were made to minimize suffering.

### Peritoneal macrophages culture

A chemical peritonitis was induced by an intra-peritoneal infusion with 3% thioglycolate (Sigma-Aldrich, St Quentin Fallavier, France) solution. Sampling was realized 72 h after injection. Peritoneal cells were collected after laparotomy, by flush out of rat peritoneal cavity with PBS without Ca^2+^ or Mg^2+^ (Fisher-Scientific, Illkirch, France) and plated in M199 culture medium (Fisher-Scientific) supplemented with 1% AB/AM (antibiotic/antimycotic) (Fisher-Scientific) and 10% FBS (Sigma-Aldrich). After 4 hours of incubation at 37°C with 5% CO_2_, nonadherent cells were removed and adherent macrophages were incubated with addition of fresh media [34].

### Islet culture

Rat islets were isolated using collagenase digestion and purification as previously described [35]. Islets were maintained in M199 culture medium supplemented with 1% Antibiotic/Antimycotic and 10% FBS at 37°C with 5% CO_2_. To study the pro-inflammatory and pro-oxidant status of islets, the experiments were performed directly after islet isolation (TO), or after 12H, 24H or 48H of culture.

### Peptides and inhibitors

Protoporphyrin IX cobalt chloride (C34H32CoN4O4Cl; CoPP; Sigma-Aldrich) was dissolved NaOH 0,1 mol/L and diluted to half in PBS. Finally, the pH was adjusted to 7.4 and the solution was sterilized by filtration. Stimulation of TLR-4 was done with 50 ng/mL lipopolysaccharide (LPS) during 24 h served as positive control. The working solution at 100 µmol/L was diluted in culture medium. CLI-095 at 1 mg/ml (C15H17CIFNO4S; InvivoGen, Toulouse, France) was dissolved in dimethylsulfoxide (DMSO, Sigma-Aldrich) and islets were treated with 1 µmole/L of CLI-095 diluted in culture medium. Islets were incubated during 24 h with the molecules immediately after isolation.

### Islet functionality

Forty islets from each experimental condition were washed extensively and incubated in Krebs Ringer bicarbonate (KRB) solution with 10% FCS (Foetal Calf Serum) and 4.4 mmol/L glucose (Sigma). Islets were then stimulated with KRB solution containing 10% FCS and 22.6 mmol/L glucose, and to finish in 4.4 mmol/L again. Each incubation step was performed for 90 min at 37°C in humidified air with 5% CO2. Supernatants were collected after each incubation time and stored at −80°C. Insulin measurements were performed using a rat insulin enzyme linked immunosorbent assay (ELISA) kit (Mercodia, Uppsala, Sweden). The results were expressed in mg/g of total protein.

### Chemotaxis assay

The chemotactic response of macrophages to rat islet supernatants was evaluated using a modified Boyden chamber according to Sigrist *et al.* [36]. The response was defined as the mean number of macrophages migrating and expressed in terms of migration index defined as the following ratio: number of macrophages attracted by the test solution (fMLP (Sigma) or culture medium conditioned by islets with or without chemoattractant)/number of macrophages attracted by the culture medium. Macrophage migration induced by culture medium was considered as random migration and was used as a negative control.

### ROS analysis

The oxidative fluorescent dye dihydroethidine (DHE, Sigma-Aldrich) was used to evaluate *in situ* formation of ROS by a method described by Dal-Ros *et al.* [37]. Unfixed pancreatic islets were cut into 4 µm-thick sections, treated with DHE (2.5 µM), incubated in a light protected humidified chamber at 37°C for 30 minutes. The level of ROS was determined using microscope and whole fluorescence of islets was quantified with the microscope software (NIS-Elements BR, Nikon Instruments Inc., Champigny-sur-Marne, France). The fluorescence intensity of the islets was quantified in fifteen arbitrarily selected islets and the mean value for each islet was calculated.

### qRT-PCR

mRNA was extracted from islets with RNeasy Plus Mini Kit and cDNA was synthesized using 500 ng RNA and a RT2 First Strand Kit, according to the manufacturer's instructions (Qiagen, Courtaboeuf, France). qRT-PCR was performed using QuantiTect SYBR Green PCR (Qiagen), and reactions were run on a My IQ 5 system (Bio-Rad, Marne la coquette, France). All primer sequences and cycle protocols were obtained from Qiagen. References were as follows (all rat specific): Ho-1 (Rn_Hmox1_1_SG QuantiTect Primer Assay, QT00175994); Cox-2 (Rn_Ptgs2_1_SG QuantiTect Primer Assay, QT00192934); Ccl2 (Rn_Ccl2_1_SG QuantiTect Primer Assay, QT00183253); Il-6 (Rn_Il6_1_SG QuantiTect Primer Assay, QT00182896); Il-10 (Rn_Il10_1_SG QuantiTect Primer Assay, QT00177618); Actin (Rn_Actb_1_SG QuantiTect Primer Assay, QT00193473); Rnr1 (Rn_Rnr1_1_SG QuantiTect Primer Assay, QT00199374) and Rplp1 (Rn_Rplp1_2_SG QuantiTect Primer Assay). Differential expression was determined by the 2^-ΔΔCT^ method [38] using three internal controls Actin, Rplp1 and Rnr1.

### Western Blot analysis

Total proteins extracts (20–40 µg) were separated on 4–12% Bis-Tris CriterionTM XT Precast Gel (Bio-Rad, Marne-La-Coquette, France) and transferred to an Immobilon PVDF membrane (Millipore, Molsheim, France). TLR-4 (mouse, 1/500; Abcam, Paris, France), HO-1-rabbit (rabbit, 1∶250; Abcam), COX-2 (rabbit, 1∶1 000; Abcam), NF-κB p65 (rabbit, 1∶5000; Cell Signaling, Danvers, USA), phospho-NF-κB p65 (Ser536, mouse, 1∶5000; Cell signaling) and β-actin (1∶8 000) primary antibodies (Abcam, Paris, France) were incubated overnight at 4°C. Anti-mouse or anti-rabbit (1/8 000, Sigma-Aldrich) horseradish peroxidase (HRP)-conjugated secondary antibody was incubated for 1 h at room temperature and developed by the LuminataTM Forte Western HRP substrate (Millipore, Molsheim, France) with Chemidoc XRS (Bio-Rad, Marne-La-Coquette, France).

### Cytokine and chemokine analyses

Islets supernatant were stored at −80°C prior to analysis. CCL2, IL-6 and IL-10 levels were determined by ELISA (R&D systems or Raybiotec, Inc., Clinisciences, Nanterre, France), according to the manufacturer's instructions.

### Endotoxin measurement

Endotoxin levels were determined on the culture medium of pancreatic islets by Limulus Amebocyte Lysate assay (Lonza, Verviers, Belgium).

### Statistical analysis

Data were expressed as mean ± SEM of the number of replicates. Differences between two groups were evaluated with t-test. Differences among three or more groups were evaluated using the nonparametric one-way ANOVA test with a Newman-Keuls multiple comparison post-test. Differences were considered significant when p<0.05.

## Results

### Islets isolation and culture exacerbate its pro-inflammatory and pro-oxidant status

**Figure 1:**
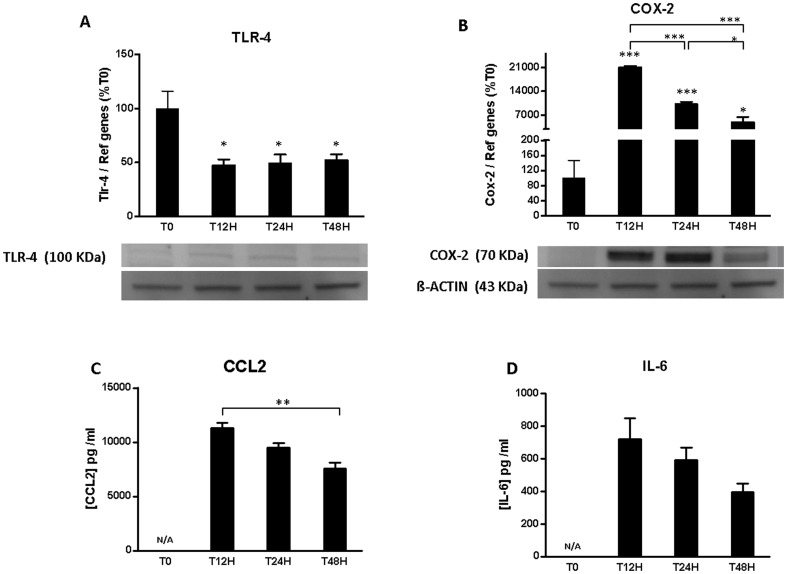
Pro-inflammatory status of pancreatic islets after isolation. Islets were incubated in M199 culture medium for 0, 12,24 and 48 h. (A,B) Gene expression of Tlr-4, Cox-2 was evaluated by qRT-PCR and protein expression of TLR-4 and COX-2 were analyzed by Western blotting. (C,D) CCL2 and IL-6 release in supernatant were evaluated by ELISA. Data shown are mean ± SEM and are representative of at least three independent experiments. *p<0.05, compared to T0, when T0 was not available (N/A) it is compared to T12H or brackets show the compared values.

Previous work showed that an inflammatory phenotype is a common feature of grafts in cell transplantation and can exacerbate deleterious pro-inflammatory response after transplantation [39]; however, the contribution of *in vitro* pre-transplantation process is well known but poorly described in the occurrence of the inflammatory phenotype. Therefore, we investigated the mRNA and protein expression of inflammatory markers on rat pancreatic islets directly after islet isolation and during 48 h of culture (Fig. 1). For each mediator, mRNA and protein expression were very well correlated. The maximum of expression of TLR-4 was obtained immediately after isolation but significantly decreased after 12 h (47±5%; p<0.05). This expression was maintained at a comparable level after 24 h and 48 h of culture (Fig. 1A). Expression of COX-2, a downstream mediator of the TLR-4 signaling pathway, was very low immediately after isolation. However, the expression was significantly increased after 12 h (p<0.001) but diminished progressively until 48 h (p<0.001) (Fig. 1B). We explored further the cytokine profile of islets *in vitro*, in particular IL-6 and CCL2, two major components involved in the islet inflammatory response [23], [39]. Similar to COX-2, IL-6 (Fig. 1C) and CCL2 (Fig. 1D) were highly released after 12 h of islet culture. This maximum amount was followed by a progressive and significant decrease of CCL-2 and IL-6 release (IL-6: 720.6 pg/mL±73.6 versus 398.1 pg/mL±21.6; CCL2: 11270.6±425.3 versus 7584.7 pg/mL±489.9).

**Figure 2:**
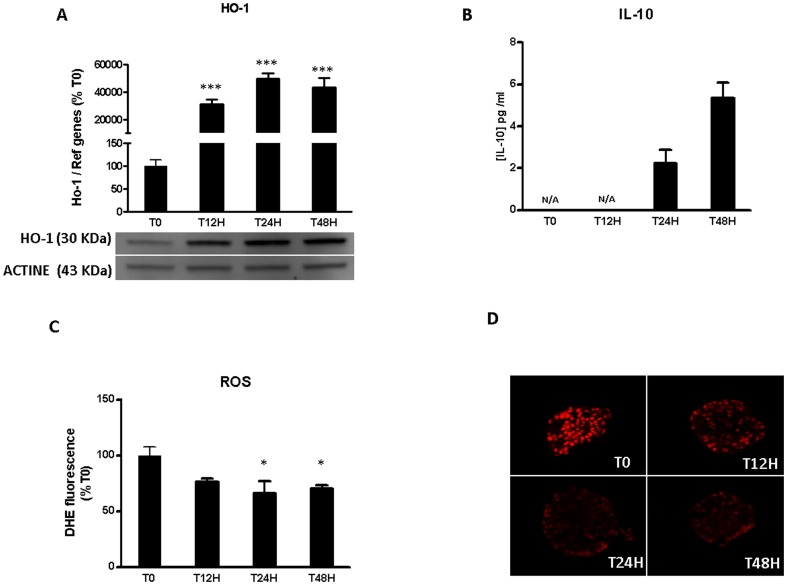
Anti-inflammatory and pro-oxidant status of pancreatic islet after isolation. Islets were incubated in M199 culture medium for 0, 12, 24 and 48 h. (A) Gene expression of Ho-1 was evaluated by qRT-PCR and protein expression of HO-1 was analyzed by Western blotting. (B) IL-10 release in supernatant was evaluated by ELISA. (C, D) ROS production was evaluated with DHE. Data shown are mean ± SEM and are representative of at least three independent experiments. *p<0,05; compared to T0 and N/A means that the values were not available.

To determine which mechanisms of defense are used by islets to protect against inflammation, we focused on HO-1 expression and IL-10 release (fig. 2). HO-1 has been demonstrated to be protective for islet by its anti-inflammatory, anti-oxidant, anti-apoptotic properties [40], [41]. On the other hand, IL-10 is an anti-inflammatory cytokine which limits the inflammatory response [42]. HO-1 was weakly expressed after isolation but significantly increased until 48 h to reach 227.7%±5.9 (fig. 2A). We observed also an increase of IL-10 during the time of culture, but the IL-10 levels remained very low. Undetectable immediately after transplantation, IL-10 secretion reached 5.3 pg/ml±0.4 after 48 h of culture (fig. 2B).

Previous data have established the link between inflammation and ROS production [43]. As a mediator of inflammation, we examined the evolution and the source of ROS during 48 h of islet culture. ROS were produced at high levels immediately after isolation, whereas during culture the production was significantly lowered (72.6%±2.7 at 48 h; p<0.05) (Fig. 2C,D).

### Islet pro-inflammatory/pro-oxidant status is TLR-4 dependent and can be limited by HO-1

To investigate whether TLR4 signaling pathways are involved in the inflammatory process *in vitro*, we treated islets in culture with an inhibitor of TLR4, CLI-095 [44], [45]. Indeed, previous work showed that islet-expressed TLR2 and TLR4 sense injury and mediate early graft failure after transplantation [31]. Given that HO-1 overexpression is known to improve islet transplantation [46]–[48] and limit the TLR signaling pathway [49], [50], we also wanted to highlight that the inflammation of islet *in vitro* is TLR-4 dependent but also that HO-1 can prevent this process by inhibiting TLR pathway activation.

**Figure 3:**
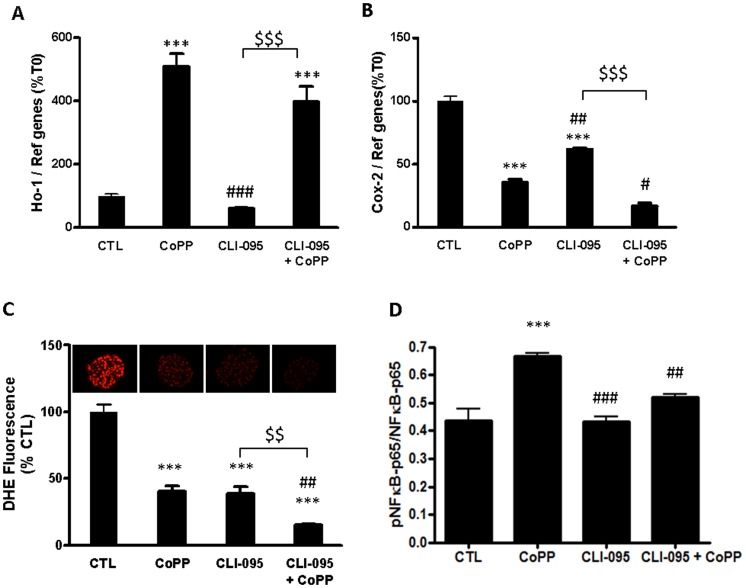
Role of TLR-4 and HO-1 on islet pro-inflammatory status. Islets were incubated for 24 h with M199 culture medium (CTL), cobalt protoporphyrin (CoPP, HO-1 inducer), CLI-095 (TLR-4 inhibitor), CoPP+CLI-095 at the same time. (A,B) HO-1 and COX-2 gene expression was analyzed by qRT-PCR. (C, D) ROS production was assessed with DHE. Data shown are mean ± SEM and are representative of at least three independent experiments. *p<0.05, in comparison with CTL. #p<0.05 in comparison with CoPP, and ^$^p<0.05 in comparison CLI-095.

Given that CoPP is known to up-regulate HO-1 expression [41], [51], we assessed whether CoPP could induce Ho-1 activation in islets. Thus, we demonstrated that CoPP alone was a powerful activator of Ho-1 (Fig. 3A) (549%±49; p<0.001), but co-treatment of islets with CoPP and the TLR-inhibitor CLI-095 also significantly increased Ho-1 activation compared to the control.

Concerning the effect of Ho-1 up-regulation on Cox-2 pathway, we observed that CoPP induced a significant decrease in Cox-2 expression (35.5%±2.6; p<0.001). We also found that CLI-095 treatment decreased Cox-2 expression (61.9±1.1; p<0.001) (Fig. 3B). The co-administration of CoPP and CLI-095 induced a greater decrease of COX-2 expression compared to the treatment with CLI-095 alone (61.9%±1.1 versus 16.8%±2.5; p<0.001).

Finally, ROS production of islets was significantly inhibited with CoPP or CLI-095 alone and co-administration of both agents improved again this inhibition (Fig. 3C).

Expression of Cox-2 is regulated by several transcription factors including nuclear NFkB [52]. Moreover, TLR4-mediated activation of the NFκB-pathway and thereby the production of IL-6 [53]. To investigate the pro-inflammatory signaling pathway involvement, we studied then the activation of NFκB(Fig. 3D). Surprisingly, CoPP induced a significant increase of NFκB phosphorylation in comparison to control islet (p<0.001). Inhibition of TLR-4 on islet alone had no effect on NFκB activation. However, co-administration of CoPP and CLI-095 significantly decreased the NFκB activation induced by CoPP (p<0.001).

**Figure 4:**
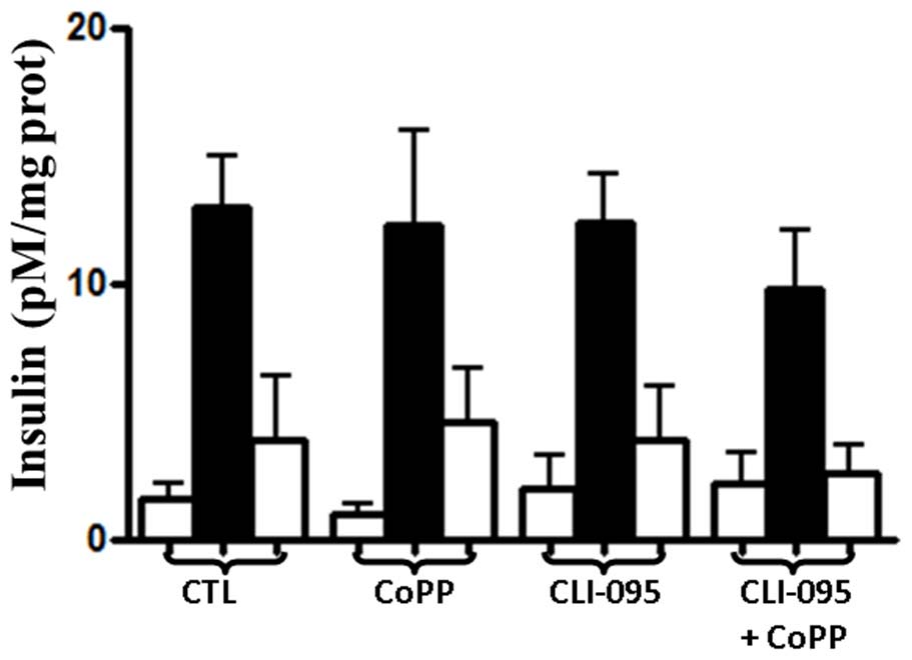
Measure of islet functionality. Islets were incubated for 24 h with M199 culture medium (CTL), cobalt protoporphyrin (CoPP, HO-1 inducer), CLI-095 (TLR-4 inhibitor), CoPP+CLI-095 at the same time. White bars: basal conditions with 4.4 mmol/L of glucose, Black bar: stimulated conditions with 22.6 mmol/L.

To confirm that islet function was not affected by the suppression of pro-inflammatory and pro-oxidant status using CLI-095 or CoPP, we studied glucose stimulation insulin released by islets. Islet functionality was maintained in basal and stimulated conditions in presence of CoPP or/and CLI-095 (figure 4).

### TLR-4 activation is inhibited by HO-1

**Figure 5:**
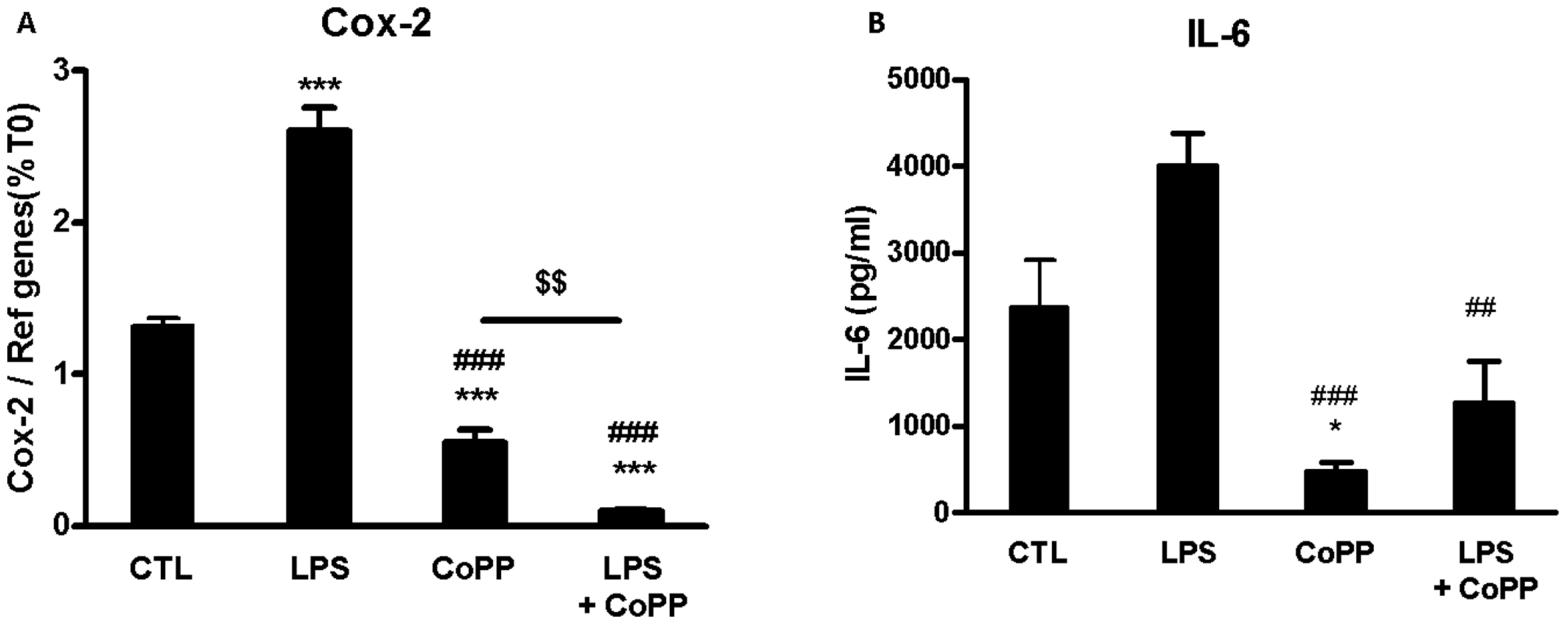
Role of HO-1 activation in TLR-4 inhibition. (A,B) Islets were incubated for 24 h with M199 culture medium (CTL), LipoPolySaccharide (LPS, TLR-4 activator), cobalt protoporphyrin (CoPP, HO-1 inducer), LPS+CoPP at the same time. (A) COX-2 gene expression was analyzed by qRT-PCR (B) IL-6 release in supernatant was evaluated by ELISA. *p<0.05, in comparison with CTL. #p<0.05 in comparison with LPS, and ^$^p<0.05 in comparison CoPP.

To establish the relationship between TLR-4 and HO-1, we determined the effect of HO-1 induction on LPS-mediated TLR-4 activation (Fig. 5). TLR-4 activation on pancreatic islets using LPS induced a significant increase of Cox-2 expression (Fig. 5A). This increase of Cox-2 expression was associated with an increase of IL-6 secretion (Fig. 5B). As previously shown figure 3, induction of HO-1 using CoPP alone on islet decreased Cox-2 expression and IL-6 secretion (p<0.001). Moreover, the pro-inflammatory status of islet induced by LPS was significantly reduced by HO-1 activation. Cox-2 expression was decreased from 2.6 to 0.5 (p<0.001) and IL-6 secretion from 4010 to 474 pg/mL (p<0.001).

### Macrophages migration is mediated by islet pro-inflammatory status

**Figure 6:**
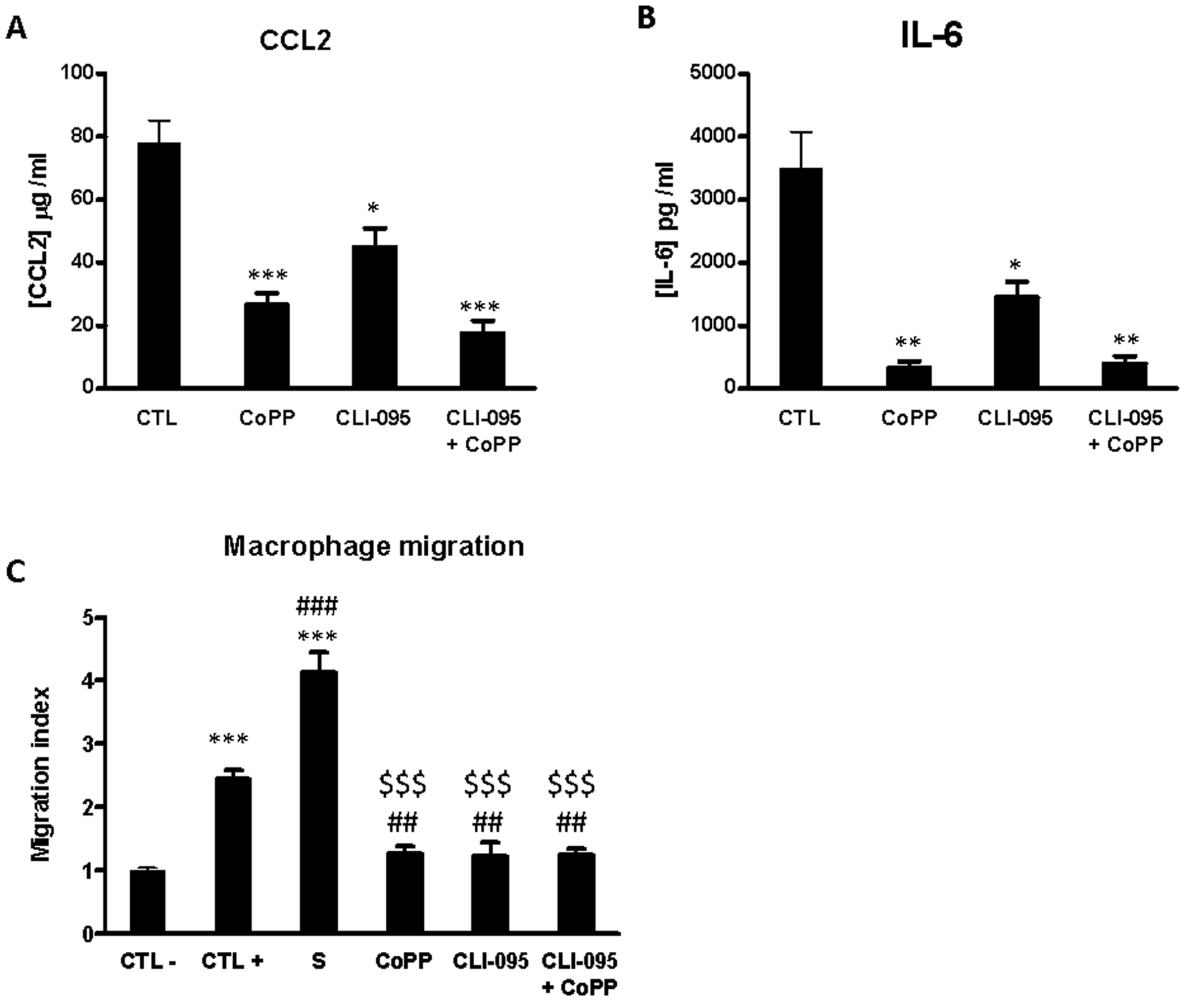
Role of TLR-4 and HO-1 on islet mediated macrophage recruitment. (A,B) Islets were incubated for 24 h with M199 culture medium (CTL), cobalt protoporphyrin (CoPP, HO-1 inducer), CLI-095 (TLR-4 inhibitor), CoPP+CLI-095 at the same time. CCL2 and IL-6 release in supernatant was evaluated by ELISA. (C) Islets were incubated for24 h with M199 culture medium (CLT−), cobalt protoporphyrin (CoPP, HO-1 inducer), CLI-095 (TLR-4 inhibitor) or CoPP+CLI-095 at the same time. Supernatant were then used to induce macrophage migration. fMLP (CTL+), non treated islet supernantant (S) were used to promote positive macrophage migration. Data shown are mean ± SEM and are representative of at least three independent experiments. A, B, C *p<0.05, in comparison with CTL; #p<0.05 in comparison with CTL+. ^$^p<0.05 in comparison with S.

Macrophages are among two major cell types infiltrating islets after transplantation [54]. In previous studies, we mimicked macrophage infiltration *in vitro* in a modified Boyden's chamber [36], [55], [56] and demonstrated that pancreatic islets release chemokines, which promote macrophage attraction, hampering engraftment of islets [36]. We hypothesized that the release of chemokines and cytokines by islets is affected by the treatment of islets with TLR-inhibitors or Ho-1 inducer. To test this hypothesis, we evaluated the effect of CoPP and CLI-095 on islet CCL-2 and IL-6 release (Fig. 6A,B). Twenty four hours treatment of pancreatic islets with CoPP induced a significantly decreased of CCL2 (26,6 µg/ml±3.7; versus 77.9 µg/ml±3.7; p<0.01) and IL-6 (77.9 µg/ml/ml±7.2 versus 3504 µg/ml±570; p<0.01) as compared to control condition. In the same way, TLR-4 inhibition resulted in a decrease of CCL-2 and IL-6 secretion. Co-administration of CoPP and CLI-095 also led to a decrease of cytokine and chemokine secretion. To confirm these results, we evaluated the impact of islets pretreatment on macrophages migration induced by islet supernatant. As expected, islet supernatant induced a massive and significant macrophage migration compared to both controls (CTL−, culture medium or CTL+, fMLP). However, with all pretreatments, islet culture supernatant lost its potency to induce macrophage migration in comparison of non-treated islet (Fig. 6C).

## Discussion

Collectively, our data indicate that the TLR signaling pathway plays an important role in the inflammatory response induced by pre-implantation injuries of pancreatic islets. Indeed, we demonstrated that cultured islets exhibit a pro-inflammatory and pro-oxidant phenotype characterized by high level of pro-inflammation markers and low defense mechanisms. Inhibition of TLR-4 confirmed a partial dependence of this inflammatory process on this signaling pathway. Moreover, HO-1 over-expression prevented the pro-inflammatory and pro-oxidant phenotype.

Our observation on COX-2 overexpression and pro-inflammatory cytokines release induced by islet culture is consistent with our previous studies [57] and others [58], [59]. The inducible isoform of cyclooxygenase, COX-2, is usually expressed at low levels in most tissues and cells, but is significantly induced by a wide range of inflammatory stimuli such as lipopolysaccharides and cytokines [60]. Some data demonstrated that COX-2 is overexpressed in pancreatic islet under both basal and stimulated conditions [61]. However, Sorli et al. have also demonstrated that preactivation of islet tissue by exposure to stimulatory agents contained in collagenase or Ficoll during islet isolation process can not alone explain this stimulation because identical treatment of hepatocytes failed to induce COX-2 gene expression [61]. Heitmeier *et al.* suggested that even if cytokines stimulate the expression of COX-2, inhibition of COX-2 activity does not protect rat and human islets from cytokine-induced beta-cell dysfunction [62]. Thus, its deleterious role on islet inflammation remains unclear because the success of *in vitro* exposure of pancreatic islets to COX-2-specific inhibitors is well known [63]. Thereby, COX-2 expression during culture is rather an indicator of pro-inflammatory pathway activation than the direct mediator of the deleterious effect of inflammation on islet.

Several studies have postulated that islets can contribute to their own death after transplantation by the elaboration of inflammatory factors [64]. Among the pro-inflammatory mediators released by islets, the pro-inflammatory cytokines plays a central role in the loss of transplanted islets by triggering their death [20]. Our results have clearly demonstrated that CCL-2 and IL-6 are strongly up-regulated in the first twelve hours after isolation in culture. In the same way, several studies have established a large release of CCL-2 [23] and IL-6 [19]. The importance of these pro-inflammatory factors has been highlighted by the correlation between their release and poor clinical outcome of islet transplants.

Furthermore, oxidative stress has also been demonstrated to play a pivotal role in islet cell damage and functional impairment. Armann et al. have demonstrated that levels of ROS in islets were correlated with the percentage of apoptotic cells and islet functional potency *in vivo* [65]. In this way, we demonstrated that the high level of ROS in islet after isolation is clearly correlated with the level of pro-inflammatory factors. Moreover, we have to keep in mind that anti-oxidative defense mechanisms in islets are very low [66] and between different species the activities of antioxidant enzymes can also vary substantially. Welsh et al. have shown that human islets have more active catalase and SOD than rodents and, consequently, are more resistant to oxidative stress [67]. Moreover, it has been clearly establish that the presence of antioxidants in isolation and preservation media detoxifies mitochondria-derived ROS and substantially improves viability of islets and their potency to normalize blood glucose in recipient diabetic animals [68]. Thus, these observations highlight that the control of ROS release must be taken into account to improve the preservation of islets *in vitro*.

Moreover, the delayed response of HO-1 and the low response of IL-10 have led us to use mediators to limit pro-inflammatory and pro-oxidant responses of islets. Our focus is due to the well accepted benefits of HO-1 on islet survival and function [40] and the fact that HO-1 mediates the anti-inflammatory effect of interleukin-10 [69]. In this way the low level of IL-10 release by islets could be induced by the up-regulation of HO-1. As suggested by several papers, in order to optimize culture conditions, enhancement of the expression of selected genes during culture that may improve islet engraftment like HO-1 [40] or IL-10 [70] is an efficient approach.

Several studies suggest that HO-1 could exert its anti-inflammatory effect by an inhibition of TLR-4 up-regulation in pancreatic islet [30]. TLR-4 is normally activated in islets during the isolation procedure, and its activation initiates inflammation, revealing the importance of the TLR-4 signaling pathway in the context of islet transplantation. Indeed, it has been demonstrated that islets express TLR-4 [31] and that TLR-4 mediates the early graft failure following islet transplantation [31], [71]. However, we highlighted the importance of this receptor even before transplantation. Given that we detected low levels of endotoxin (lower than 0.7 EU/ml), induction of the TLR signaling pathway was due to a “sterile inflammation”. More precisely, it has been demonstrated that TLRs can bind endogenous ligands, called Damage-Associated Molecular Patterns (DAMPs). Thus, these ligands can be generated by injury during pancreas conservation, islet isolation or hypoxia during the time of culture. Some studies have suggested that proteins generated by stressed islets like high mobility group box 1 (HMGB1) or residues of digested extracellular matrix like heparin sulfate [72], could be at the origin of this sterile inflammation [73]. The identity of these factors could be investigated in future studies.

Our results also showed that simultaneous inhibition of TLR-4 and induction of HO-1 are equally efficient in blocking COX2, CCL2 and IL-6 activation as inhibition of TLR4 alone. Hence, our data suggest that HO-1 is an upstream regulator of the TLR4 signaling pathway. In fact, HO-1 activation induced NFκB activation. Several studies have already demonstrated that during the inflammatory process, the increased of HO-1 expression was correlated was the activation of NFκB [74]. However, while TLR-4 inhibition has no effect on NFκB activation induced during islet culture, co-treatment of islet with HO-1 activator and TLR-4 inhibitor significantly increased NFκB activation induced by CoPP. These data suggested that HO-1 and TLR-4 could act in a unique signaling pathway to induce the pro-inflammatory status of islet.

To confirm this hypothesis, we studied the effect of HO-1 induction on TLR-4 activation using LPS. We then demonstrated that TLR-4 activation was completely inhibited by HO-1 activation confirmed the relationship between HO-1 and TLR-4 signaling pathway on islet pro-inflammatory status.

However, the signaling pathway involved in the production ROS seems to be different. Indeed, we observed a higher inhibition of ROS production with the simultaneous TLR-4 inhibition and HO-1 activation. Our data suggest that ROS are generated by two distinct pathways dependent on either HO-1 or TLR-4. Thus, we have demonstrated that even if the regulation of TLR-4 is dependent on HO-1 in the case of inflammatory mediator generation such as COX-2, IL-6 and CCL2, it seems that these signaling pathways act in an independent manner to regulate the production of ROS.

In order to show the benefits brought by the inhibition of the TLR-4 signaling pathway, we compared the recruitment of peritoneal macrophages after induction with islet supernatant. These cell culture supernatants were harvested 24 h after treatment of islet. We demonstrated that the macrophage chemotaxis was reduced by islet supernatant as previously described [56]. The decrease of macrophages migration with TLR-4 inhibition and HO-1 activation could be explained by the decreased levels of secreted CCL-2. Our data are in agreement with the study of Piemonti et al. suggesting that CCL-2 is the major chemokine for monocytes in the supernatants of cultured pancreatic islets [22].

In conclusion, we demonstrated that TLR-4 signaling pathway plays a crucial role in the regulation of the pro-inflammatory and pro-oxidant response of islet *in vitro*. It appears that pro-inflammatory responses induced by TLR-4 can be inhibited by HO-1 while the pro-oxidant response induced by TLR-4 seemed independent. These data demonstrate unambiguously the importance of the TLR-4 signaling pathway in deleterious islet-inflammation and characterize the well-known benefits of HO-1 on islet transplantation and may help to target new therapeutic molecules that could protect islets before transplantation.
